# Brain Metastases from Primary Cardiac Tumors: A Systematic Review of Diagnosis, Treatment, and Prognosis

**DOI:** 10.3390/cancers17101621

**Published:** 2025-05-10

**Authors:** Salvatore Marrone, Ignazio Alessio Gueli, Roberta Lo Coco, Lorenzo Scalia, Salvatore Rizzica, Giuliana Baiamonte, Roberta Costanzo, Antonino Salvatore Rubino, Gianluca Ferini, Giuseppe Emmanuele Umana, Gianluca Scalia

**Affiliations:** 1 Department of Neurosurgery, Sant’Elia Hospital, 93100 Caltanissetta, Italy; 2 Cardiology Division, Fondazione Toscana Gabriele Monasterio, 56124 Pisa, Italy; igueli@ftgm.it; 3 Unit of Pathology, Sant’Elia Hospital, 93100 Caltanissetta, Italy; 4 Division of Cardiology, Umberto I Hospital, 94100 Enna, Italy; 5 Department of Internal Medicine, Sant’Elia Hospital, 93100 Caltanissetta, Italy; 6 Electric, Electronics and Computer Engineering Department, University of Catania, 95125 Catania, Italy; 7 Department of Neurosurgery, Villa Sofia Hospital, 90146 Palermo, Italy; 8 Department of Medicine and Surgery, University of Enna Kore, 94100 Enna, Italy; antoninosalvatore.rubino@unikore.it (A.S.R.); umana.nch@gmail.com (G.E.U.); gianluca.scalia@outlook.it (G.S.); 9 Cardiac Surgery Unit, C.C.D. “G.B. Morgagni”, Heart Center, 95030 Pedara, Italy; 10 Department of Radiation Oncology, REM Radioterapia Srl, 95126 Viagrande, Italy; 11 Department of Neurosurgery, Trauma and Gamma Knife Center, Cannizzaro Hospital, 95126 Catania, Italy; 12 Neurosurgery Unit, Department of Head and Neck Surgery, Highly Specialized Hospital of National Importance “Garibaldi”, 95124 Catania, Italy

**Keywords:** cardiac tumors, brain metastases, cardiac myxoma, cardiac sarcoma, multimodal imaging

## Abstract

**Background**: Primary cardiac tumors (PCTs) are rare entities, with only a minority being malignant and capable of distant dissemination. Among the rarest and most challenging metastatic events are brain metastases originating from cardiac tumors. Due to the heart’s direct access to systemic circulation, even benign tumors such as atrial myxomas may cause cerebral embolic phenomena. Understanding the distinct biological behavior, diagnostic pathways, therapeutic strategies, and prognostic implications of these cases remains limited by the scarcity of the available literature. **Methods**: A systematic review was conducted following PRISMA guidelines. PubMed, Scopus, Embase, and other major databases were systematically searched using specific MeSH terms and keywords related to cardiac tumors and brain metastases. After applying strict inclusion and exclusion criteria, nineteen studies were included, comprising sixteen single-patient case reports and three multi-patient series. Extracted data included tumor histology, cardiac and brain imaging findings, neurological presentation, treatment approaches, and patient outcomes. **Results**: A total of 320 patients were analyzed. Atrial myxomas represented the predominant benign tumors causing embolic cerebral events, while angiosarcomas and other cardiac sarcomas were responsible for true hematogenous brain metastases. Brain involvement was frequently hemorrhagic and manifested with seizures, focal deficits, or signs of intracranial hypertension. Cardiac echocardiography and cardiac magnetic resonance imaging (CMR) were essential for tumor detection, while brain MRI, including SWI and DWI sequences, and CT scanning were critical for cerebral lesion characterization. Treatment strategies varied according to tumor type and included surgery, radiotherapy, and systemic therapies. Malignant cardiac tumors correlated with a poor prognosis, with median survival post-CNS involvement ranging from 12 to 14 months. **Conclusions**: Brain metastases from PCTs, though rare, represent a distinct and serious clinical phenomenon. Benign tumors like myxomas mainly cause embolic cerebral events, whereas malignant tumors, particularly sarcomas, lead to true metastatic brain lesions. Recognizing this biological distinction is crucial for diagnosis, prognostication, and therapeutic planning. An integrated multidisciplinary approach combining advanced cardiac and neuroimaging techniques is vital for early detection and appropriate management. Despite multimodal treatment, survival remains limited, underscoring the urgent need for novel targeted therapies and improved surveillance strategies.

## Simple Summary

Primary cardiac tumors (PCTs) are extremely rare, and brain involvement arising from such tumors represents an even more exceptional event. In this systematic review, we analyzed 19 studies comprising 320 patients to investigate the diagnostic challenges, imaging findings, therapeutic approaches, and clinical outcomes associated with brain metastases of cardiac origin. We emphasize the distinction between benign tumors, such as atrial myxomas, which primarily cause embolic cerebral events, and malignant tumors, such as sarcomas, which demonstrate true hematogenous dissemination to the brain. Understanding this biological divergence is crucial for optimizing diagnostic pathways, therapeutic decisions, and prognostic evaluation. Although survival remains poor in malignant cases, early diagnosis and multidisciplinary management may contribute to improved patient outcomes.

## 1. Introduction

Primary cardiac tumors (PCTs) are rare, representing a small fraction of intracardiac masses, which are more commonly secondary to metastatic spread from extracardiac malignancies [1]. Although often benign, primary cardiac tumors deserve clinical attention due to their potential for malignant transformation and systemic dissemination [2,3]. Approximately 80% are benign, with myxomas being the most common, while 20% are malignant, predominantly sarcomas [1,3,4]. These tumors arise from various cardiac structures, including the myocardium, fibrous connective tissue, conduction system, endocardium, or subpericardial vessels. Tumors originating from serosal surfaces, such as mesotheliomas, involve the pleura and pericardium and are outside the scope of this review [5]. While benign cardiac tumors, particularly atrial myxomas, often result in embolic phenomena causing ischemic or hemorrhagic cerebral events, malignant tumors such as cardiac sarcomas possess true hematogenous metastatic potential, leading to multifocal brain involvement. This biological distinction has critical implications for diagnosis, clinical management, and prognosis. Although the blood–brain barrier (BBB) offers protection, malignant cardiac tumors can metastasize to the brain by disrupting barrier integrity through inflammatory mediators [6]. Cerebral involvement is often presented as hemorrhagic, multifocal lesions with disproportionate edema, affecting eloquent and non-eloquent brain areas [7]. Clinically, cardiac tumors can manifest through hemodynamic compromise or acute structural failure, while asymptomatic cases may be detected incidentally [3,8]. Advances in non-invasive imaging, particularly echocardiography and cardiac MRI, have significantly improved early diagnosis and follow-up [9,10]. Benign tumors are typically amenable to surgical resection, but malignant or high-grade tumors often require multimodal treatment, including chemotherapy and radiotherapy [9,11,12]. Although prognosis remains poor for high-grade malignancies, early detection and intervention can significantly improve outcomes by preventing metastatic progression [13,14].

## 2. Materials and Methods

### 2.1. Study Design

**Figure 1:**
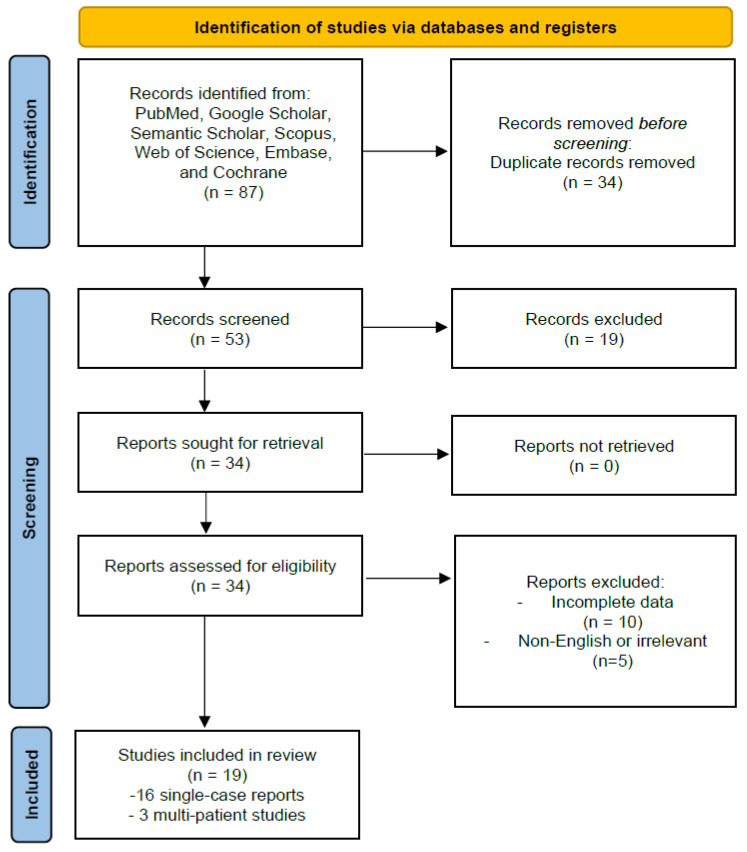
PRISMA 2020 flow diagram illustrating the study selection process. Out of 87 initially identified records, 34 duplicates were removed. After screening 53 unique studies, 19 were excluded based on title/abstract. Of the 34 full-text articles assessed for eligibility, 15 were excluded for incomplete or irrelevant data. Nineteen studies were included in the final analysis (sixteen case reports and three multi-patient series).

This systematic review adheres to the Preferred Reporting Items for Systematic Reviews and Meta-Analyses (PRISMA) 2020 guidelines (Figure 1). The objective of this study is to provide a comprehensive synthesis of existing knowledge, assess the prevalence and characteristics of brain metastases originating from PCT, and examine the clinical implications of these metastases.

### 2.2. Literature Search Strategy

An extensive search was conducted on PubMed, Google Scholar, Semantic Scholar, Scopus, Web of Science, Embase, and Cochrane Library. Search terms included “cardiac tumors and brain metastases”, “heart neoplasms and CNS metastases”, “cardiac sarcoma and cerebral metastases”, and related combinations to encompass different tumor types and neurological implications. Initially, 87 articles were identified. After removing duplicates and applying screening criteria—including relevance to cardiac tumors with cerebral involvement, the completeness of clinical data, and language—19 studies were included in the final analysis following full-text evaluation.

### 2.3. Eligibility Criteria

**Inclusion criteria**: Studies were eligible if they were published in a peer-reviewed journal, investigated cerebral metastases in patients with primary cardiac tumors, included at least five patients or, in the case of exceptionally rare conditions, provided detailed single-patient case reports that offered novel clinical or pathological insights, confirmed the presence of brain metastases through clinical presentation, radiological evidence, or histopathological analysis, used imaging modalities such as magnetic resonance imaging (MRI), Computed Tomography (CT), or Positron Emission Tomography (PET) to detect cerebral involvement, and were published in English.

**Exclusion criteria**: Studies were excluded if they involved non-human experimental models, focused solely on extracranial metastatic spread without evaluating brain involvement, were based on single-case reports lacking sufficient diagnostic or therapeutic detail, consisted of unpublished theses, meeting abstracts, editorials, or other non-peer-reviewed materials, did not provide clear clinical or imaging confirmation of cerebral metastases, or were duplicates of previously included studies.

### 2.4. Data Extraction and Synthesis

Data were extracted on study design, sample size, tumor type, brain metastasis detection methods, clinical outcomes, and treatments. Emphasis was placed on understanding the biological behavior of cardiac tumors with cerebral dissemination and evaluating therapeutic impacts on survival and quality of life.

### 2.5. PRISMA Framework

**Figure 2:**
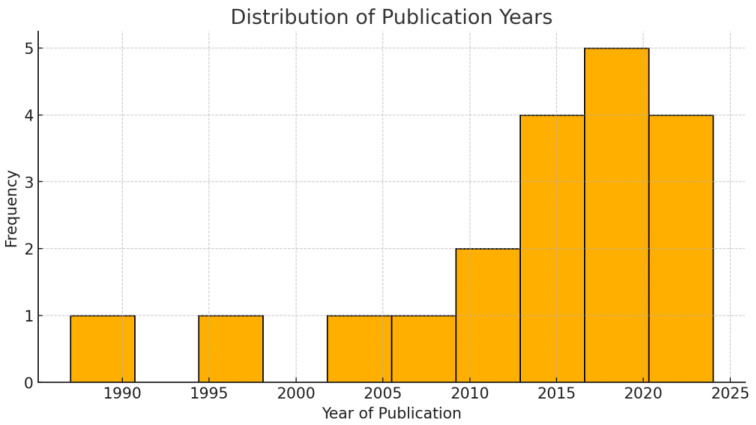
Timeline distribution of the included studies. Most publications on this rare topic have emerged in the last decade.

A PRISMA flow diagram was used to illustrate study selection, ensuring transparency in the identification, screening, eligibility assessment, and inclusion process (Figure 2).

## 3. Results

A detailed evaluation of the 19 included studies reveals substantial heterogeneity in both the number of patients and the types of cardiac tumors investigated. Sixteen of these references comprise single-patient case reports, whereas three describe larger cohorts, including 47 patients in the study by Brinjikji et al. [13], 218 patients in the multicenter SEER analysis by Guan et al. [15], and 39 patients in the series reported by Siontis et al. [8] (Table 1). Although these combined papers represent 320 patients in total, any attempt at overarching statistical generalization must consider the markedly disparate sample sizes and the differences in the histopathological profiles that emerge from benign versus malignant cardiac neoplasms. Nonetheless, an aggregated descriptive and inferential analysis offers valuable insights into the demographic characteristics, clinical presentation, tumor histology, treatment approaches, and prognostic outcomes associated with brain metastases arising from primary cardiac tumors.

### 3.1. Demographic Data

**Figure 3:**
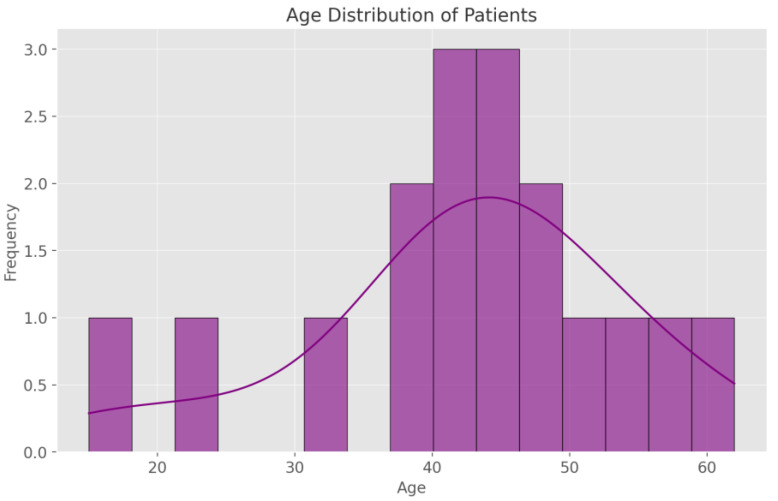
Age distribution of patients with cardiac tumors associated with brain involvement. Most patients are diagnosed between the ages of 40 and 50.

A closer look at the 16 single-patient case reports reveals a mean age of 42 years (range: 15–62) and a median of 42.5 years, underscoring the fact that individuals can present with cardiac tumor-related cerebral lesions from adolescence through later adulthood. Among these sixteen cases, nine patients (56%) were female and seven (44%) were male, indicating a relatively balanced sex distribution in this subset. By contrast, the multi-patient studies provide a broader demographic perspective. Siontis et al. [8] included 39 individuals whose median age was 41 years (range: 2–77), with 67% female (26 patients) and 33% male (13 patients). The sample analyzed by Guan et al. [15] (218 patients) generally skewed toward those under the age of 65, but a detailed breakdown of sex was not supplied in the summarized data. Brinjikji’s investigation [13] of 47 cases of cardiac myxoma did not provide a comprehensive sex distribution; however, the illustrative example featured in that report involved a 50-year-old female. Collectively, these data demonstrate that patients with cardiac tumor-associated central nervous system (CNS) involvement vary considerably in age and sex, thereby emphasizing the broad epidemiologic reach of this phenomenon (Figure 3).

### 3.2. Clinical Presentation and Symptomatology

Across both single-patient and multi-patient accounts, two primary clinical patterns emerged: some patients initially exhibited cardiac symptoms such as palpitations, dyspnea, or pericardial effusion, while others presented directly with neurological complaints, including intracranial hemorrhage, seizures, focal deficits, or persistent headaches. In single-patient reports, approximately half had neurological symptoms as their first presentation. A subset of cases reflected systemic embolization, particularly associated with atrial myxomas. In larger cohorts, Siontis et al. [8] reported that 31% of patients developed brain metastases, frequently presenting with hemorrhage or seizures. Similarly, Guan et al. [15] observed that CNS involvement correlated with poorer survival, suggesting neurological symptoms as an indicator of advanced disease.

### 3.3. Histopathological Distribution

**Figure 4:**
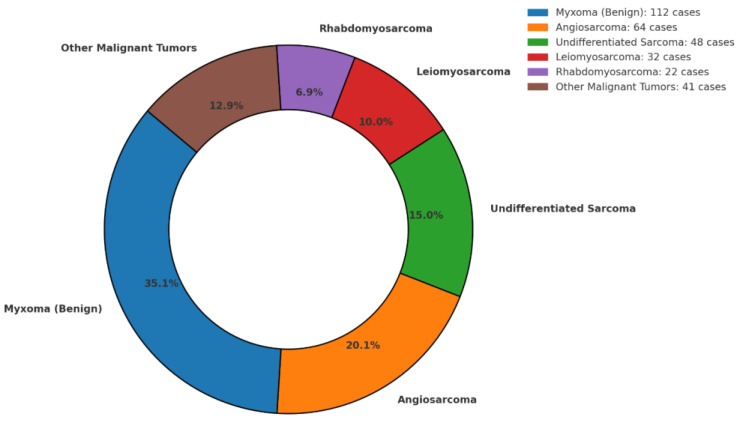
Histopathological distribution of cardiac tumors: Myxomas were the most frequent benign tumors, followed by angiosarcomas and undifferentiated sarcomas among malignant lesions.

**Figure 5:**
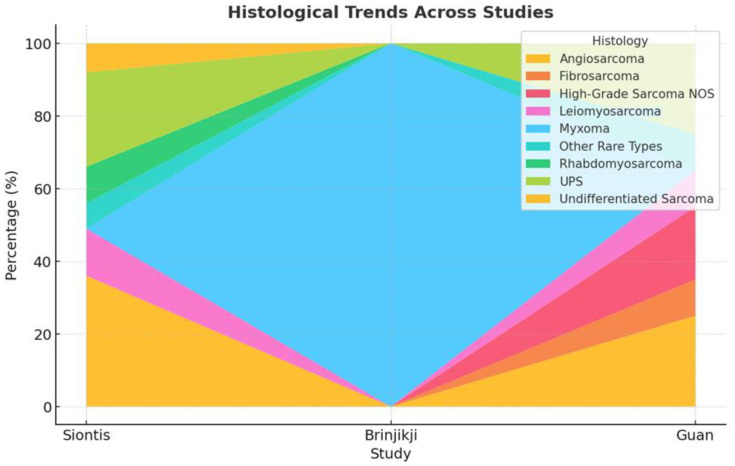
Comparative histological breakdown of cardiac tumors across the three largest included studies (Brinjikji [13], Guan [15], Siontis [8]). Myxomas dominated Brinjikji’s cohort, while angiosarcomas and undifferentiated sarcomas were prevalent in the Guan and Siontis series.

Atrial myxomas emerged as the most frequently encountered benign lesion, especially in case reports, where they accounted for most single-patient descriptions. These benign tumors often originated in the left atrium and, despite their non-malignant nature, produced substantial morbidity through embolic or hemorrhagic events in the brain. On the other hand, malignant tumors—particularly angiosarcomas, high-grade undifferentiated pleomorphic sarcomas, leiomyosarcomas, and rhabdomyosarcomas—prevailed in the more extensive series. Siontis et al. [8] documented angiosarcoma as the predominant entity in 36% of their 39 patients, whereas Guan et al. [15] focused on an array of sarcomas within a 218-patient sample. These high-grade neoplasms typically exhibited a marked propensity for distant metastases, culminating in multifocal intracranial lesions in a significant subset of patients (Figure 4 and Figure 5).

### 3.4. Location of Cardiac Tumors and Propensity for Metastasis

**Figure 6:**
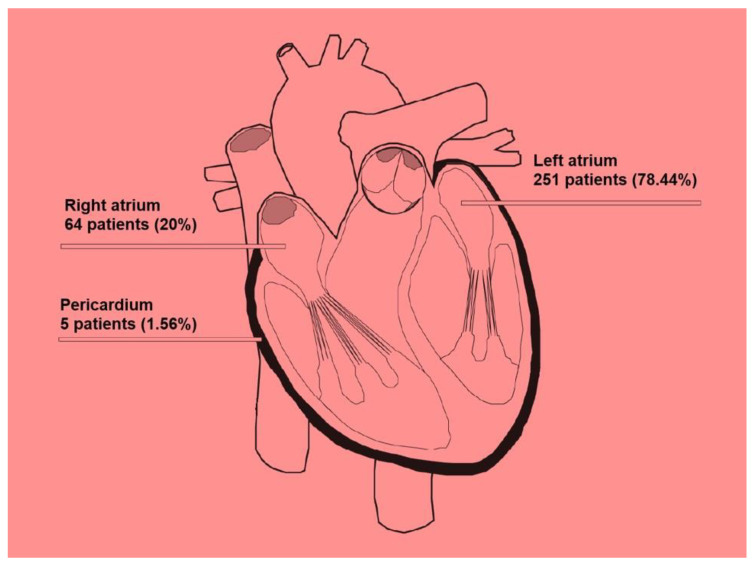
Anatomical distribution of cardiac tumors: The left atrium is the most frequently involved site (78.44%), followed by the right atrium (20%) and pericardium (1.56%).

Among benign tumors, the left atrium predominated as the primary intracardiac site of origin, consistent with the classical localization of atrial myxomas on the interatrial septum. However, right atrial involvement was also observed, particularly for angiosarcomas, which occasionally advanced to produce widespread metastatic deposits. Siontis et al. [8] documented that 46% of their cases involved tumors in the left atrium, 41% affected the right atrium, and 13% were in the pericardium, a distribution that has direct implications for clinical presentation and systemic spread. Although quantitative comparisons of metastatic rates between left- and right-sided tumors remain limited, the existing literature suggests that left-sided lesions, especially myxomas, may favor cerebral embolization, whereas right-sided malignant tumors (e.g., sarcomas) may initially produce lung metastases but can also breach systemic circulation (Figure 6).

### 3.5. Neurological Manifestations and Lesion Characteristics

The hemorrhagic transformation of brain lesions was a predominant finding across case reports and cohorts. Approximately 60–70% of single-patient cases described intracranial hemorrhage, often presenting acutely with headache, vomiting, or focal neurological deficits. Lesions were commonly located in the frontal, parietal, or occipital lobes, with multifocal disease and cerebellar involvement also reported. In Siontis et al. [8], about one-third of patients experienced hemorrhagic or ischemic events requiring urgent intervention. Although not all studies specified lesion sites, a consistent pattern of hemorrhagic complications necessitating neurosurgical consultation was evident.

### 3.6. Therapeutic Strategies

The therapeutic approach typically combined cardiac surgery, neurosurgical intervention, and adjuvant therapies such as radiotherapy or systemic chemotherapy, tailored to the histological type of the primary tumor. In the majority of benign myxoma cases, the surgical excision of the cardiac mass was both feasible and associated with a favorable prognosis, provided that the intracranial lesions were either resected or controlled via radiotherapy. Among single-case examples, postoperative follow-up extended to 1–4 years without documented recurrence in several individuals. By contrast, patients with malignant cardiac sarcomas faced a more challenging clinical course. Siontis et al. [8] reported that 25 of their 39 patients (64%) underwent surgical resection of the primary tumor, whereas 28 (72%) received chemotherapy. Radiotherapy was performed in 11 cases (26%), commonly to address CNS metastases. The authors noted a modest but statistically significant extension of median overall survival, from 12.1 months in the entire series to approximately 14 months in those who underwent surgery plus systemic therapy. Guan’s study from the SEER database [15] conveyed that the mean survival hovered around 3.64 years but was significantly reduced in the subset of patients who progressed to brain metastases, although a precise hazard ratio was not consistently detailed across all subgroup analyses. Meanwhile, Brinjikji et al. [13] underscored the importance of vigilant, and often long-term, neurovascular follow-up in myxoma cases, given the propensity for new or recurrent vascular anomalies even after cardiac tumor resection.

### 3.7. Prognosis and Follow-Up

**Figure 7:**
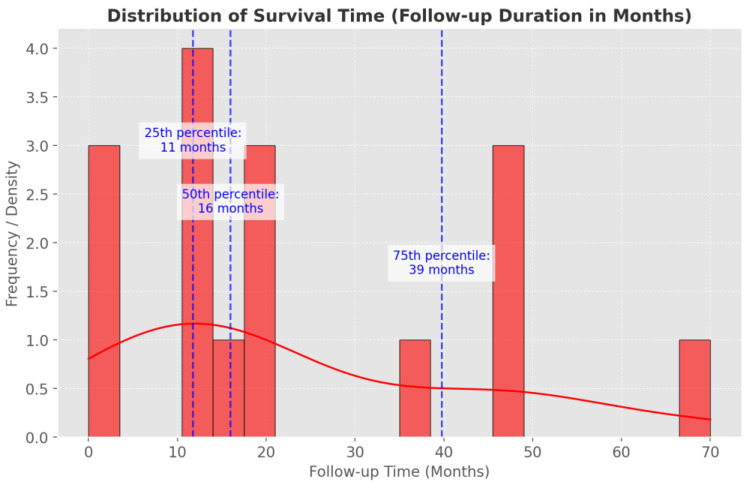
Distribution of patient survival time after diagnosis. The median follow-up duration was 16 months. The interquartile range (IQR) for survival was 11 to 39 months, meaning that 25% of patients had a survival of 11 months or less, while 75% survived up to 39 months.

Prognostic outcomes vary substantially based on tumor histology, the burden of intracranial disease, and the availability of complete surgical resection. Benign myxomas appeared to offer relatively more favorable trajectories, with some case reports documenting 2–4 years of uneventful follow-up post-resection. By contrast, high-grade sarcomas, particularly angiosarcomas, often demonstrated fulminant progression and early mortality; in one case, death ensued within 70 days despite aggressive intervention [26]. Siontis et al. [8] highlighted that the development of brain metastases strongly correlated with shortened survival, a conclusion parallel to Guan’s larger-scale evaluation [15], in which intracranial involvement emerged as a key adverse prognostic indicator. Although multimodality treatment (surgery, chemotherapy, and radiotherapy) yielded a discrete survival advantage in some cohorts, the inherent aggressiveness of advanced sarcomas rendered relatively uncommon long-term remission (Figure 7).

## 4. Discussion

Brain metastases commonly arise from primary tumors of the breast, prostate, kidney, and colon, with a predilection for the cortico–subcortical junction due to vascular distribution. Radiologically, these lesions exhibit marked edema, necrosis, and peripheral contrast enhancement, often requiring differential diagnosis with infections and primary neoplasms. Neurological symptoms typically appear late, triggered by mass effect or increased intracranial pressure. Brain involvement from cardiac malignancies, although extremely rare, presents similarly. Sarcomas, especially angiosarcomas, demonstrate aggressive behavior with frequent hemorrhagic transformation [30,31]. Uniquely, benign atrial myxomas can also seed the brain via tumor emboli, producing hemorrhagic lesions and vascular abnormalities like pseudoaneurysms [32,33,34]. These lesions often retain a relatively well-delineated, non-infiltrative appearance, reflecting their slower biological evolution compared to high-grade malignancies [35].

### 4.1. Pathways of Spread

Several mechanisms are hypothesized to explain the development of brain involvement in patients with cardiac tumors, including hematogenous dissemination, where malignant tumor cells enter systemic circulation directly from the heart and reach cerebral vessels, bypassing pulmonary filtration [36]; tumor embolization, particularly in the case of atrial myxomas, where friable tumor fragments detach and embolize to cerebral arteries, causing ischemic or hemorrhagic complications [37]; paradoxical embolism through a patent foramen ovale, which enables embolic material to bypass pulmonary capillaries and reach systemic circulation; and the subsequent disruption of the blood–brain barrier (BBB), where circulating tumor cells alter local vascular permeability through inflammatory or angiogenic mediators, creating a permissive microenvironment for the metastatic colonization of the brain parenchyma [7,38,39].

### 4.2. Histopathology Relevant to Brain Dissemination

Among benign cardiac tumors, myxomas are most relevant for brain dissemination due to their embolic potential. They are highly vascularized, with a myxoid stroma, and show calretinin positivity. Detached tumor emboli can reach the cerebral circulation, leading to ischemic or hemorrhagic events. Among malignant tumors, angiosarcomas exhibit aggressive hematogenous dissemination linked to their vascular nature and endothelial marker expression (CD31, CD34, ERG) [40]. Their structural fragility predisposes them to intracranial hemorrhage. Undifferentiated pleomorphic sarcomas (UPS), characterized by high mitotic rates and extensive necrosis, also have strong metastatic potential, including to the brain [41,42,43]. In contrast, cardiac fibromas, papillary fibroelastomas, rhabdomyomas, lipomas, and hemangiomas rarely influence cerebral dissemination due to their lower vascularity or lack of aggressive behavior. Primary cardiac lymphomas, notably, diffuse large B-cell lymphomas (DLBCLs), can involve the brain secondarily via hematogenous spread, particularly in the context of aggressive disease courses [44].

### 4.3. Heterogeneity in Tumor Histology and Metastatic Patterns

Atrial myxomas are the most frequently reported benign cardiac tumors associated with brain involvement, mainly through tumor embolization rather than true metastasis [45,46,47]. These cases often manifest as cerebral infarctions or hemorrhagic events, underlining their emboligenic behavior [19,27,48,49]. In contrast, malignant cardiac tumors, particularly sarcomas, demonstrate true hematogenous dissemination to the CNS, frequently resulting in multifocal brain lesions [7,8,16,18,23]. Among them, angiosarcomas [39] and osteogenic sarcomas [38] show especially aggressive patterns, with a predilection for hemorrhagic brain metastases. Interestingly, the location of the primary cardiac tumor appears to influence the metastatic pathway. Left atrial tumors, such as myxomas, primarily cause cerebral embolization, whereas right atrial malignancies, including angiosarcomas and rhabdomyosarcomas, often metastasize first to the lungs before reaching the brain [8,16,20]. Data from larger cohorts [8,13,15] reinforce the concept that right-sided cardiac tumors follow a distinct, sequential metastatic route compared to left-sided tumors.

### 4.4. Imaging Features of Brain Metastases and Cardiac Tumors

#### 4.4.1. Brain Imaging

Magnetic resonance imaging (MRI) remains the cornerstone for diagnosing brain metastases, offering superior soft tissue contrast and lesion characterization [50,51]. Critical sequences include susceptibility-weighted imaging (SWI), which is highly sensitive for detecting hemorrhages [52], and diffusion-weighted imaging (DWI) coupled with apparent diffusion coefficient (ADC) mapping, essential for identifying ischemic lesions and differentiating high-grade tumors [51]. Gadolinium-enhanced MRI plays a pivotal role in assessing blood–brain barrier disruption and leptomeningeal spread. Computed tomography (CT) retains a complementary role, particularly in acute settings, for detecting hemorrhages and calcifications [52,53]. The addition of SWI phase-filtered imaging assists in distinguishing hemorrhagic from calcific components, a differentiation that standard imaging may fail to resolve [54].

#### 4.4.2. Cardiac Imaging

The imaging evaluation of cardiac tumors begins with transthoracic (TTE) and transesophageal echocardiography (TEE), providing initial structural and functional assessment [52]. CMR offers unparalleled tissue characterization, differentiating benign from malignant masses through the analysis of morphology, vascularization, and infiltration patterns [53]. Gadolinium-enhanced sequences, particularly early (EGE) and late gadolinium enhancement (LGE), provide critical insights into tumor viability and fibrosis. CT is reserved for anatomical delineation and the detection of calcifications [47], while 18F-FDG PET/CT is instrumental for metabolic assessment, staging, and therapeutic monitoring, particularly in highly aggressive neoplasms [53]. Imaging modalities are presented in Table 2, ordered according to clinical relevance.

### 4.5. Polyneoplastic Syndromes Involving Both Systems

Certain genetic syndromes, including Carney complex, neurofibromatosis types 1 and 2 (NF1 and NF2), and tuberous sclerosis complex (TSC), are associated with the development of both cardiac and cerebral tumors. Carney complex, inherited in an autosomal dominant fashion, is characterized by multiple neoplasms, particularly cardiac myxomas [55,56]. These highly vascular tumors can embolize, leading to cerebral ischemic events or metastatic-like lesions. This disorder is linked to mutations in the PRKAR1A gene, causing dysregulated cell proliferation. NF1, caused by mutations in the NF1 gene, results in the development of neurofibromas and carries a lifetime risk of malignant transformation into peripheral nerve sheath tumors (MPNSTs) between 8% and 13% [56]. Cardiac involvement, although rare, has been reported. NF2, due to NF2 gene mutations, is distinguished by bilateral vestibular schwannomas and the occurrence of other CNS tumors such as meningiomas and ependymomas [56]. Cardiac tumors are not a typical manifestation. TSC results from mutations in TSC1 or TSC2 genes and leads to benign tumor formation in various organs, including cardiac rhabdomyomas and CNS lesions like cortical tubers [57,58,59,60,61]. Cardiac rhabdomyomas, common in children with TSC, generally regress spontaneously and do not metastasize. While Carney complex and TSC demonstrate tumorigenic potential in both heart and brain, true metastatic spread from cardiac tumors to the brain remains exceedingly rare. Their underlying pathophysiology involves genetic mutations promoting multisystemic tumor proliferation.

### 4.6. Treatment Strategies and Prognostic Considerations

Initial management typically begins with corticosteroid therapy to reduce vasogenic edema, alleviate mass effect, and stabilize neurological symptoms prior to definitive interventions [33,62]. Antiepileptic drugs are recommended when lesions are near eloquent cortical areas. Surgical treatment is considered for solitary, large (>2 cm) brain metastases accessible at the cortical surface, particularly when systemic disease is controlled and the Karnofsky Performance Status (KPS) exceeds 70% [12,63,64,65,66]. Complete or gross total resection, ideally supramarginal (>5 mm beyond contrast enhancement margins), is the goal to reduce recurrence and mass effect [67,68]. En-bloc resection is preferred when feasible to minimize leptomeningeal dissemination [69]. Urgent decompressive surgery is indicated for posterior fossa lesions causing acute intracranial hypertension [70]. However, surgery is generally contraindicated for patients with multiple (>4) lesions, poor KPS, or extensive systemic disease (Recursive Partitioning Analysis class III) [71,72]. Focused ultrasound has emerged as a promising adjunct by enhancing blood–brain barrier permeability and potentiating immunotherapeutic delivery [70,71]. Chemotherapy and systemic therapies are generally limited by the blood–brain barrier’s selective permeability but can be considered based on the chemosensitivity profile of the primary cardiac tumor [59,60]. Immune checkpoint inhibitors combined with stereotactic radiosurgery have shown potential to improve survival, although careful monitoring for radionecrosis is essential [72]. Radiotherapy remains a cornerstone. Stereotactic radiosurgery (SRS) is preferred for 1–4 brain metastases, focusing high-dose radiation to minimize damage to adjacent parenchyma [71,72]. Whole-brain radiotherapy (WBRT) with hippocampal sparing is considered when there are multiple (>3) metastases with bilateral hemispheric involvement [70,71,72]. In the context of cardiac tumors, treatment strategies must recognize the fundamental differences between benign embolic phenomena and true malignant brain metastases. For atrial myxomas, surgical excision remains curative in most cases, with long-term favorable outcomes [15,31], although late neurological sequelae, including delayed embolism or vascular anomalies, warrant prolonged follow-up [15]. For cardiac sarcomas, multimodal therapy combining surgery, radiotherapy, and chemotherapy is essential, but prognosis remains dismal. Median survival after CNS involvement ranges from 12 to 14 months, even with aggressive treatment [8,16,34]. Targeted therapies and biomarkers for cardiac tumor-related brain metastases remain an unmet need and an area of future research [8,16]. This review emphasizes that cerebral complications in cardiac tumors differ depending on whether the mechanism is embolic (benign myxomas) or metastatic (malignant sarcomas) [19,27,31,33,40]. Hemorrhagic risk, especially in angiosarcomas, remains a major clinical challenge [8,20]. Despite surgical and systemic advancements, survival outcomes for malignant cardiac tumors with brain dissemination remain poor, reinforcing the need for innovative therapeutic approaches and vigilant surveillance strategies.

## 5. Future Perspectives

Future research should focus on identifying specific biomarkers capable of predicting the risk of cerebral dissemination in patients with PCTs. Early detection through biomarker integration with advanced neuroimaging techniques, such as MRI with susceptibility-weighted imaging (SWI) and diffusion-weighted imaging (DWI), could significantly optimize surveillance and therapeutic planning [41,42,43,44]. Given the differing biological behaviors between benign and malignant cardiac tumors, future surveillance protocols should be stratified accordingly:

- Patients with benign atrial myxomas should undergo long-term neurological follow-up, aimed at detecting delayed embolic events or vascular complications even years after tumor resection [13,31].
- Patients with malignant tumors, such as cardiac sarcomas, require intensive systemic and cerebral imaging to identify early multifocal metastatic spread and guide prompt, aggressive multimodal therapy [8,15].

Advances in cardiac surgery, including the use of modern bioprosthetic valves with superior hemodynamic performance and reduced thromboembolic risk [73], will facilitate safer tumor resections and minimize neurological complications [74]. In selected cases with massive cardiac infiltration, the use of total artificial hearts, such as SynCardia, may enable more radical surgical approaches followed by systemic oncologic treatments [75]. In parallel, new strategies, such as targeted systemic therapies capable of crossing the blood–brain barrier, focused ultrasound to enhance therapeutic delivery, and immunotherapy, are promising avenues to improve outcomes for patients with brain metastases secondary to cardiac tumors [62,76,77]. Ultimately, a multidisciplinary management model involving cardiologists, neurosurgeons, oncologists, and radiologists will be essential to optimize treatment strategies and improve survival and quality of life in these complex cases.

## 6. Conclusions

Brain metastases originating from PCTs represent a rare but clinically significant entity, characterized by heterogeneous biological behavior depending on the tumor histotype. While benign tumors such as myxomas primarily cause embolic cerebral events, malignant cardiac sarcomas demonstrate true hematogenous metastatic potential, often leading to multifocal and hemorrhagic brain involvement. Early diagnosis through advanced multimodal imaging and timely multidisciplinary intervention are crucial to improving outcomes. Despite surgical and systemic therapeutic advances, the prognosis for patients with brain dissemination from cardiac malignancies remains poor. Recognizing the fundamental difference between benign embolic phenomena and malignant metastatic disease is crucial: patients with benign tumors benefit from vigilant, long-term neurological surveillance, whereas malignant cases require aggressive multimodal oncological management. Future efforts must focus on developing predictive biomarkers, enhancing blood–brain barrier-penetrant therapies, and refining integrated management strategies to better address this challenging clinical scenario.

## Figures and Tables

**Table 1:** Summary of individual and aggregate data extracted from the included studies, detailing patient demographics, tumor histology, cardiac tumor location, presence of brain metastases, and treatment outcomes.

Author	Year	Patient Number	Gender/ Age	Clinical Presentation	Histological Type	Cardiac Topography	Brain Symptoms	Brain Topography	Treatment	Follow-Up
Geyer SJ et al. [16]	1987	1	M/23	Cough; pericardial effusion	RMS	RA	Pulmonary sx	NS	NS	NS
Dan S et al. [17]	1997	1	F/32	Right hemiparesis; TIA	OsteoS	LA	Generalized	Brain	Atrial recon; valve repl	18 mo
Altundag MB et al. [18]	2005	1	F/41	Palpitations; HA; vertigo; seizures	Myx	LA	HA; vertigo; seizures	Cerebrum; cerebellum	Surg; WBRT (30 Gy)	48 mo, NED
Ikeya E et al. [19]	2006	1	M/49	SOB; tamponade	AS	RA	Hemorrhage; hemiparesis	RT lobe	Pericardiotomy; symptomatic	Died, 37 days
Idris B et al. [20]	2012	1	F/15	HA; nausea; vomiting	Myx	LA	HA; vomiting	R parietal; occipital	Craniotomy	12 mo, NED
Radoi MP et al. [21]	2012	1	M/45	HA; gait issues; seizures	Myx	LA	Intra-cr. Tumors; aneurysms	Parietal; frontal	Surgery	12 mo; NED
Pasalic D et al. [14]	2013	1	F/45	HA; mental changes; somnolence	UPS	LA	Multiple lesions	Frontal; parietal	Radiosurg; atrial surg	Progression
Kierdaszuk B et al. [22]	2014	1	F/41	Limb weakness	Myx	LA	Hemorrhagic lesions	Parietal; occipital	Surgery	18 mo; stable
Brinjikji W et al. [13]	2015	47	Mixed/50	Arm weakness; visual changes	Myx	LA	Infarcts; aneurysms	Multifocal cortical	Surg; conservative	Long-term; mild deficits
Rose D et al. [23]	2016	1	M/44	Light-headed; speech disturbance	Myx	LA	Aneurysms	4th ventricle	WBRT; steroids	14 mo, died
Badaloni F et al. [12]	2017	1	M/41	Partial seizures; hemiparesis	MyxoS	Mitral valve; LA	Seizures; aphasia	Parietal, rolandic; cerebellum	Craniotomy	Died; 70 days
Roque A et al. [24]	2019	1	F/48	HA; fatigue; numbness	Myx	LA	Hemorrhage; pseudoaneurysms	Parietal, frontal, occipital	WBRT; HS	18 mo; stable
Siontis BL et al. [8]	2019	39	Mixed/41	Dyspnea; CP; edema	AS, others	LA (46%), RA (41%)	Brain mets (31%)	Multifocal	Surg; CT; RT	Median OS 12.1 mo
Wan Y et al. [25]	2019	1	F/39	HA; blurred vision; vomiting	Myx	LA	Hemorrhage; aneurysms	MCA, ACA terr.	Craniotomy, clip	11 mo; recovery
Maas JA et al. [26]	2020	1	M/62	TIA; arm numbness; vision	Myx	LA	Hemorrhage; edema	R parieto-occipital	Brain surg	48 mo; NED
Guan T et al. [15]	2022	218	Mixed/Var.	Cardiac mass sx	Sarcoma	LA/RA	Rare	Parenchyma	Surg (some)	Poor if brain mets
Shah Z et al. [27]	2023	1	M/37	Neuro sx; hemorrhage	AS	RA	Hemorrhagic lesions	R frontal lobe	Radiosurg; systemic	12 mo; died
Basu A et al. [28]	2024	1	F/56	HA; arm weakness; vision	Myx	LA	Hemorrhagic lesions	Multiple sites	WBRT (20 Gy/5 fx)	48 mo; recovery
Solís-Gómez R et al. [29]	2024	1	F/54	Dizziness; seizures	Myx	LA	Visual sx; seizures	Occipital; multiple	Surg; valve repl; craniotomy	3 yr; NED

**Abbreviations:** ACA = Anterior Cerebral Artery, AS = angiosarcoma, BSx = Brain Symptoms, BT = Brain Topography, CP = clinical presentation, CTopo = Cardiac Topography, CT = computed tomography, fx = Fractions, FU = follow-up, HA = headache, HT = histological type, HS = hippocampal sparing, LA = left atrium, MCA = Middle Cerebral Artery, Met = metastases, MRI = magnetic resonance imaging, Myx = myxoma, MyxoS = myxofibrosarcoma, NED = No Evidence of Disease, NS = Not Specified, OS = overall survival, PEff = pericardial effusion, PET/CT = positron emission tomography/CT, RA = right atrium, RMS = rhabdomyosarcoma, SRS = stereotactic radiosurgery, SOB = Shortness of Breath, TIA = Transient Ischemic Attack, UPS = undifferentiated pleomorphic sarcoma, and WBRT = Whole Brain Radiotherapy.

**Table 2:** A summary of key imaging characteristics of common cardiac tumors (benign, malignant, secondary, and pseudotumors) using echocardiography, CMR, CT, and PET/CT.

Tumor Type	Echocardiography (TTE/TEE)	Cardiac Magnetic Resonance (CMR)	Computed Tomography (CT)	Positron Emission Tomography/CT (PET/CT)
Myx	Mobile mass LA (fossa ovalis); isoechoic	Hyperintense SSFP/T2; iso T1; hetero CE	Well-defined; ±calcifications; no CE	Mild/absent uptake
Rhabdo	Hyperechoic; intramural or protruding	Iso/hyper SSFP/T2; iso T1; no CE	Homogeneous; hypodense	Mild/absent
Lip	Hyperechoic (intracav); hypoechoic (pericard)	Hyper SSFP/T1; hypo T2; no CE	Homogeneous; hypodense	Mild/absent
Fibro	Small, mobile, and valve-attached mass	Hypo SSFP/T1/T2; no CE	Hypodense mass	None
Hema	Mass from myocardium or pericardium	Hyper SSFP/T2; iso/hetero T1; CE+	Well-defined margins	Mild/absent
AS	Irreg., immobile, infiltrative RA mass, and PEff	Iso/hyper SSFP; hetero T1; hyper T2; strong CE	Infiltrative; into pericardium/cavity	High
RMS	Immobile and infiltrative mass	Iso/hyper SSFP; hetero T1; hyper T2; CE+	Infiltrative/protruding mass	High
DLBCL	‘Cauliflower-like’ and all chambers	Iso/hyper SSFP; hetero T1; hyper T2; CE+	Hypodense; irreg/well-defined	High
Met	Multiple lesions and often PEff	Iso/hyper SSFP; hetero T1; hyper T2; CE+	Hypodense; CE varies	High (aggressive dep.)
Thrombus	Hyperechoic, akinetic zones, and no CE	Hypo SSFP/T1; hyper T2; no CE	Filling defect; no CE	None

**Abbreviations:** AS = angiosarcoma, CE = contrast enhancement, CMR = cardiac magnetic resonance, CT = Computed Tomography, DLBCL = diffuse large B-cell lymphoma, Hema = hemangioma, LA = left atrium, Met = Cardiac Metastases, PET/CT = Positron Emission Tomography/Computed Tomography, RA = right atrium, Rhabdo = rhabdomyoma, RMS = rhabdomyosarcoma, SSFP = Steady-State Free Precession, and TTE/TEE = transthoracic/transesophageal echocardiography.
